# Inter-observer agreement in athletes ECG interpretation using the recent international recommendations for ECG interpretation in athletes among observers with different levels of expertise

**DOI:** 10.1371/journal.pone.0206072

**Published:** 2018-11-21

**Authors:** S. Schneiter, L. D. Trachsel, T. Perrin, S. Albrecht, T. Pirrello, P. Eser, B. Gojanovic, A. Menafoglio, M. Wilhelm

**Affiliations:** 1 Department of Cardiology, Bern University Hospital (Inselspital), Bern, Switzerland; 2 Swiss Federal Institute of Sports, Swiss Olympic Medical Center, Magglingen, Switzerland; 3 La Tour Sport Medicine, Swiss Olympic Medical Center, Hôpital de La Tour, Geneva, Switzerland; 4 Clinic for Cardiology, Ospedale San Giovanni, Bellinzona, Switzerland; University of Miami School of Medicine, UNITED STATES

## Abstract

**Introduction:**

International criteria for the interpretation of the athlete’s electrocardiogram (ECG) have been proposed. We aimed to evaluate the inter-observer agreement among observers with different levels of expertise.

**Methods:**

Consecutive ECGs of Swiss elite athletes (≥14 years), recorded during routine pre-participation screening between 2013 and 2016 at the Swiss Federal Institute of Sports were analysed. A medical student (A), a cardiology fellow (B) and an electrophysiologist (C) interpreted the ECG’s independently according to the most recent criteria. The frequencies and percentages for each observer were calculated. An inter-observer reliability analysis using Cohen Kappa (κ) statistics was used to determine consistency among observers.

**Results:**

A total of 287 ECGs (64.1% males) were analysed. Mean age of the athletes was 20.4±4.9 years. The prevalence of abnormal ECG findings was 1.4%. Both, normal and borderline findings in athletes showed moderate to good agreement between all observers. κ scores for abnormal findings resulted in excellent agreement (κ 0.855 in observer A vs C and B vs C to κ 1.000 in observer A vs B). Overall agreement ranged from moderate (κ 0.539; 0.419–0.685 95% CI) between observer B vs C to good agreement (κ 0.720; 0.681–0.821 95% CI) between observer A vs B.

**Conclusions:**

Our cohort of elite athletes had a low prevalence of abnormal ECGs. Agreement in abnormal ECG findings with the use of the recently published International recommendations for ECG interpretation in athletes among observers with different levels of expertise was excellent. ECG interpretation resulted in moderate to good overall agreement.

## Introduction

There is an ongoing controversy regarding the addition of a uniform resting 12-lead electrocardiogram (ECG) in the cardiovascular pre-participation screening (PPS) strategy of competitive athletes between Europe and the U.S. [1, 2] A high rate of false-positive findings with additional costs of downstream cardiologic work-up [3] and insufficient inter-observer agreement in the interpretation of the athlete’s ECG among physicians remain major barriers. [4, 5]. Since the original European proposition of standardized criteria for the interpretation of the ECG in athletes 2010 recommended by the European society of cardiology (ESC), several modifications have been published to improve specificity without compromising sensitivity. [6–8] Recently, based on a convention of a group of International experts modern standardized recommendations in this field have been co-published. [9–11] Furthermore, several recent studies found a reduction in the false-positive rates of athlete ECGs using standardised criteria as compared to ‘usual’ interpretation with a cost reduction using more recent criteria. [3, 12, 13]

To the best of our knowledge, no study examined the inter-observer agreement for ECG interpretation in athletes based on the most recent International recommendations in observers with different levels of expertise so far. The aim of our pilot investigation was to assess the inter-individual observer agreement in athletes ECG interpretation between a medical student (no training, no expertise), a cardiology fellow (in training, little expertise) and an experienced electrophysiologist, the latter acting as a reference regarding training and expertise. We hypothesised that, using the most recent International ECG criteria, a medical student and a cardiology fellow would be able to detect the clinically relevant ECG abnormalities (high sensitivity) with a low number of false-positive findings (high specificity).

## Methods

### Study population

From April 2013 to February 2016, consecutive 12-lead resting ECGs from Swiss Elite athletes were recorded at the Swiss Olympic Medical Center (SOMC) in Magglingen (Federal Institute of Sports, Switzerland) during routine pre-participation examination. According to the Swiss society of sports medicine this evaluation is recommended to be initiated from age 14, and to be repeated every one to two years until the end of the sports career.[14] All athletes were asymptomatic and competed on national or international level. Exclusion criteria were known cardiovascular disease, a recent history of underperformance, abnormal tiredness, acute infectious disease or cardiac symptoms, as well as abnormal physical examination. In case of more than one ECG recordings during the study period, only the first ECG was considered for analysis. The resting 12-lead ECGs were recorded with a Schiller Cardiovit AT-10 automat (voltage 10mm/mV, paper speed 25mm/s) based on current recommendations. [15] All ECGs were anonymized, coded, scanned in high resolution and transferred to the University Clinic for Cardiology at the Bern University Hospital.

### Observers

The ECGs were provided digitally to three different observers: Observer A (SS) was a medical student shortly before achieving his medical doctor (MD) degree. At that time he had neither expertise nor training in the interpretation of athletes ECGs. Before starting the analysis of the ECGs he was instructed by two experienced sports cardiologists from the University Hospital Bern (LDT, MW) according to the most recent International recommendations for the ECG interpretation in athletes. [9–11]. Observer B (TP) was an advanced cardiology fellow certified in sports medicine with little expertise while on training (during a fellowship for sports cardiology). [16] Observer C (AM) was a senior cardiologist with several years of expertise in electrophysiology and sports cardiology. Observer C had > 2 years expertise and ≥ 1000 routine athletes ECGs evaluated, while observers A and B had not [17]. For the purpose of this study, the ECGs were analysed retrospectively.

### ECG Interpretation

All three observers were provided with a basic spreadsheet (Microsoft Excel, Santa Rosa, CA) in which they entered RR interval, P-wave duration, PR interval, QRS interval, QT interval, QRS-axis and T-axis. When present, computer-generated measurements made by Schiller Cardiovit AT-10 ECG Measurement and Interpretation Software (Version April 2002) were used for analysis. If not obviously normal, they were measured with manual calipers. The QT and QTc Interval were measured with the tangent method in lead II or V5 if computer-generated measurements were abnormal (e.g. Long QT/Short QT).[18] The Bazett’s formula was used for heart rate correction of the QT interval.[19] The ECGs were ‘independently’ analysed point by point according to the most recent International recommendations by all three observers. [9–11] After completion of the analyses, the three databases were merged with a second coded spreadsheet containing information on age, sex, race, and training volume. The ECGs were finally classified into four groups: ECGs with no findings, ECGs with findings normal in athletes, ECGs with borderline findings, and ECGs with abnormal findings. ECGs with one borderline finding were reclassified as normal in athletes, ECGs with two or more borderline findings were reclassified as abnormal findings. [9–11] For each observer (A, B and C) the frequencies and percentages of ECGs classified as no, normal in athletes, borderline, and abnormal ECG findings were calculated.

### Statistical analysis

All statistical analyses were performed using SPSS Statistics for Windows, version 23, (IBM Corporation, Armonk, NY). The results are presented as means +/- standard deviation or counts (percentages). *P*-values of less than 0.05 were considered significant. Confidence intervals (CI) were defined as 95%. Data for each observer are reported as frequencies and percentages. An inter-observer reliability analysis using the Cohen κ (kappa) statistics was performed to determine consistency among observers and to correct the percentage of agreement. κ (kappa) scores between 0.01–0.20 were classified as none to slight, 0.21–0.40 as fair, 0.41–0.60 as moderate, 0.61–0.80 as good (substantial), and 0.81–1.00 as almost perfect agreement. [20] Each pair of observers was compared (A vs B, A vs C and B vs C) by Cohen κ (kappa), a measure of agreement that is corrected for random agreement. [20]

### Ethics

This study was approved by the Ethics committee of the Canton of Bern, Switzerland. The study protocol complies with the Declaration of Helsinki. All enrolled athletes signed an informed consent form allowing the scientific use of their ECGs and clinical data.

## Results

A total of 390 ECGs were recorded. Two-hundred and eighty-seven ECGs were considered for the final analysis (exclusion of 60 athletes who were aged < 14 years, 34 with follow-up ECGs of already included athletes, and 9 who were referees). Included athletes were 64.1% male and the mean age was 20.4±4.9 years. The prevalence of abnormal ECG findings ranged from 1% (observer C) to 1.4% (observer A, B). Baseline characteristics of all includes athletes are shown in Table 1.

**Table 1:** Athlete characteristics.

Characteristics	n = 287
Age (years)	20.4 ± 4.9
Male gender	184 (64.1)
Race (Caucasian)	286 (99.7)
Body mass index (kg/m^2^)	22.4 ± 4.9
Training duration (years)	8.7 ± 4.9
Average weekly training time (hours)	17.7 ± 7.1
Level of competition	
International	237 (82.6)
National	50 (17.4)

Data are shown as mean ± SD or frequency (proportion).

### ECG classification

**Fig 1:**
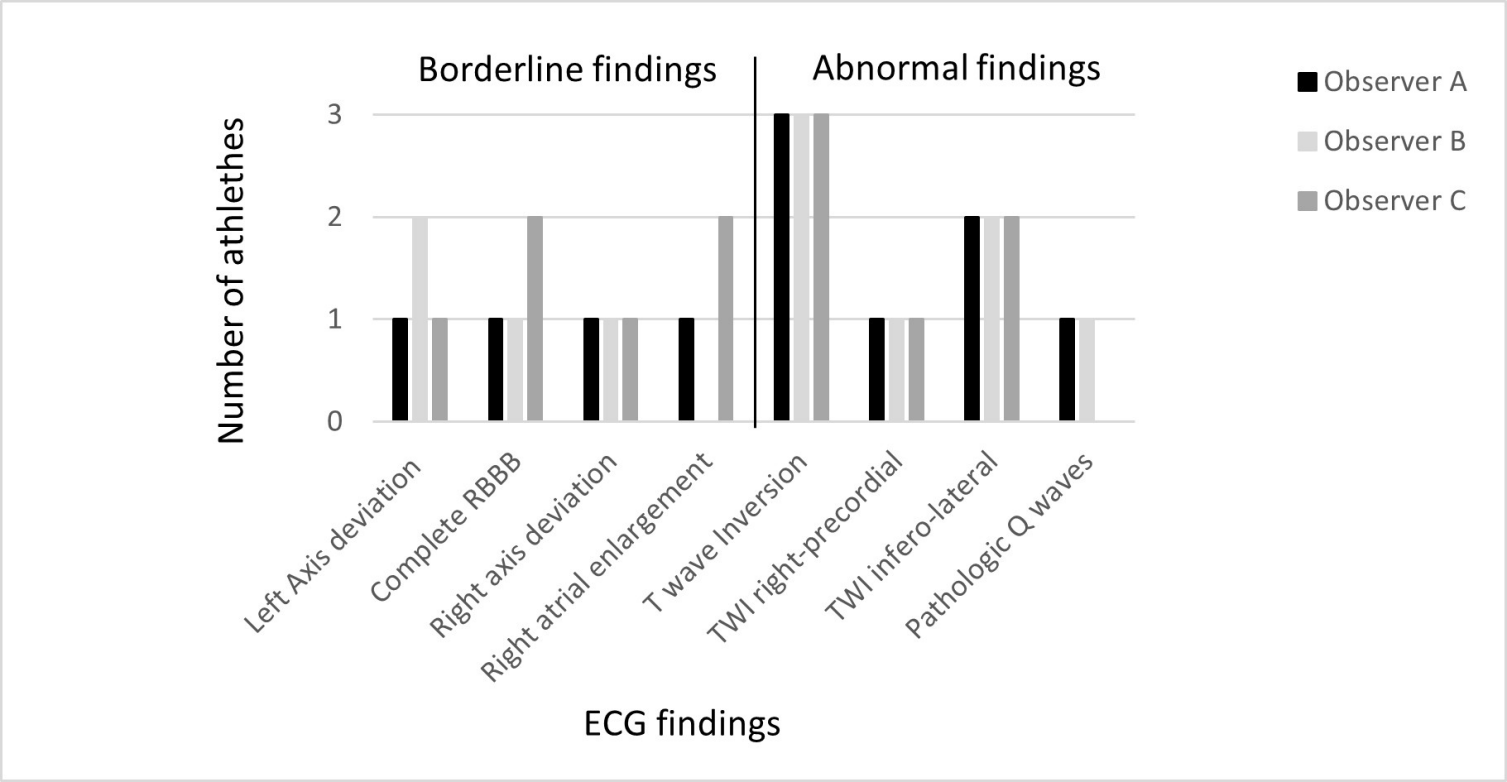
Agreement for borderline and abnormal ECG findings according to the recent International criteria in the same athletes. RBBB, right bundle branch block; TWI, T-wave Inversion.

The proportion and frequency of athletes with no ECG findings ranged from 46 (15%) in observer B to 64 (22%) in observer C. The ECGs classified as normal in athletes ranged from 217 in observer C (75.6%) to 233 in observer B (81.2%). A lower number of athletes was classified with early repolarization in observer A (n = 34) as compared to observer B (n = 58) and observer C (n = 65). There was no athlete classified with junctional escape rhythm, Mobitz type 1 second-degree AV block, T-wave inversion (TWI) in leads V1- V3 ≤ 16 years and convex ST segment elevation combined with TWI in leads V1- V4 among all three observers. Borderline findings were classified as such only if present in isolation. No ECG was classified as abnormal with two or more borderline criteria. Observer A and B classified the same 4 athletes as having abnormal findings (3 with TWI, 1 with pathological Q-wave). Observer C classified the same 3 athletes as the other observers with TWI as abnormal findings (2 with infero-lateral TWI, 1 with right precordial TWI). One athlete with a Q-wave duration > 40ms was correctly categorized as abnormal by observer A and B, but not C. The ECG of this 24 year old cyclist revealed a Q-wave duration of 46–50 ms in lead V4-V6 measured with manual calipers. Agreements on borderline and abnormal ECG findings in the same athletes are given in Fig 1.

### Inter-observer agreement

The resulting frequencies, percentages and calculated κ scores are summarized in Table 2. Agreement for no ECG findings ranged from moderate (κ 0,553; 95% CI 0,431–0,674) in observer B vs C to good (κ 0,705; 95% CI 0,597–0,813) in observer A vs B. Inter-observer agreement for findings normal in athletes ranged from moderate (κ 0,549; 95% CI 0,431–0,666) in observer B vs C to good (κ 0.723; 95% CI 0,623–0,823) in observer A vs B. For borderline findings, calculated κ scores ranged from moderate (κ 0.493; 95% CI 0,065–0,920) between observer B vs C to good (κ 0.747; 95% CI 0,407–1,000) between observers A vs C. κ scores for abnormal findings ranged from excellent in observer A vs C and B vs C (κ 0.855; 95% CI 0,515–1,000 and κ 0.855; 95% CI 0,574–1,000, respectively) to excellent in observer A vs B (κ 1.000). Overall agreement according to the recent International recommendations was between 85% and 91% with moderate agreement (κ 0.539; 95% CI 0,419–0,658) between observer B vs C to good agreement (κ 0.720; 95% CI 0,618–0,821) between observer A vs B. Agreement for no, normal in athletes, abnormal and overall agreement was best between observers A vs B except for borderline findings.

**Table 2:** Frequencies, percentages and calculated κ scores of agreement between observers (A vs B, A vs C, B vs C) on the presence of no, normal in athletes, borderline and abnormal ECG findings and overall agreement according to the recent International criteria.

Observers	A vs B		A vs C		B vs C	
ECG findings	Agreement	Kappa (95% CI)	Agreement	Kappa (95% CI)	Agreement	Kappa (95% CI)
**No findings**	**39 (91,6%)**	0,705 (0,597–0,813)*	**45 (89,2%)**	0,676 (0,570–0,781)*	**35 (86,1%)**	0,553 (0,431–0,674)*
**Normal in athletes ECG**	**215 (90,9%)**	0,723 (0,623–0,823)*	**203 (88,1%)**	0,669 (0,567–0,770)*	**203 (84,6%)**	0,549 (0,431–0,666)*
-Sinus bradycardia	112 (99.3%)	0,985 (0,965–1,000)*	112 (97,9%)	0,956 (0,921–0,991)*	110 (97,2%)	0,942 (0,902–0,981)*
-Sinus arrhythmia	157 (85,7%)	0,699 (0,615–0,783)*	123 (81,9%)	0,643 (0,558–0,991)*	125 (76,6%)	0,546 (0,457–0,634)*
-Ectopic atrial rhythm	9 (97.5%)	0,707 (0,501–0,912)*	6 (97,9%)	0,658 (0,402–0,912)*	6 (97,5%)	0,621 (0,364–0,877)*
-First-degree AV block	3 (99,3%)	0,747 (0,407–1,000)*	4 (99,6%)	0,887 (0,667–1,000)*	3 (99,6%)	0,855 (0,574–1,000)*
-Incomplete RBBB	43 (94,8%)	0,820 (0,731–0,908)*	40 (93%)	0,729 (0,625–0,833)*	35 (93%)	0,736 (0,626–0,845)*
-Isolated QRS voltage criteria LVH	19 (93%)	0,618 (0,465–0,771)*	23 (95,1%)	0,740 (0,610–0,832)*	15 (93%)	0,562 (0,389–0,734)*
-Early repolarisation	27 (86.8%)	0,514 (0,382–0,645)*	30 (85,4%)	0,533 (0,409–0,656)*	46 (89,1%)	0,680 (0,618–0,814)*
**Borderline ECG**	**2 (99.3%)**	0,663 (0,224–1,000)*	**3 (99,6%)**	0,747 (0,407–1,000)*	**2 (98,6%)**	0,493 (0,065–0,920)*
**Abnormal ECG**	**4 (100%)**	1*	**3 (99,3%)**	0,855 (0,574–1,000)*	**3 (99,3%)**	0,855 (0,515–1,000)*
Overall agreement**	**261 (90,9%)**	0,720 (0,618–0,821)*	**253 (88,2%)**	0,663 (0,559–0,767)*	**243 (84,7%)**	0,539 (0,419–0,658)*

* p < 0.001,

** Overall agreement for all included athlete ECG’s, n = 287

AV block, atrio-ventricular block; CI, confidence interval; RBBB, right bundle brunch block; LVH, left ventricular hypertrophy.

**Fig 2:**
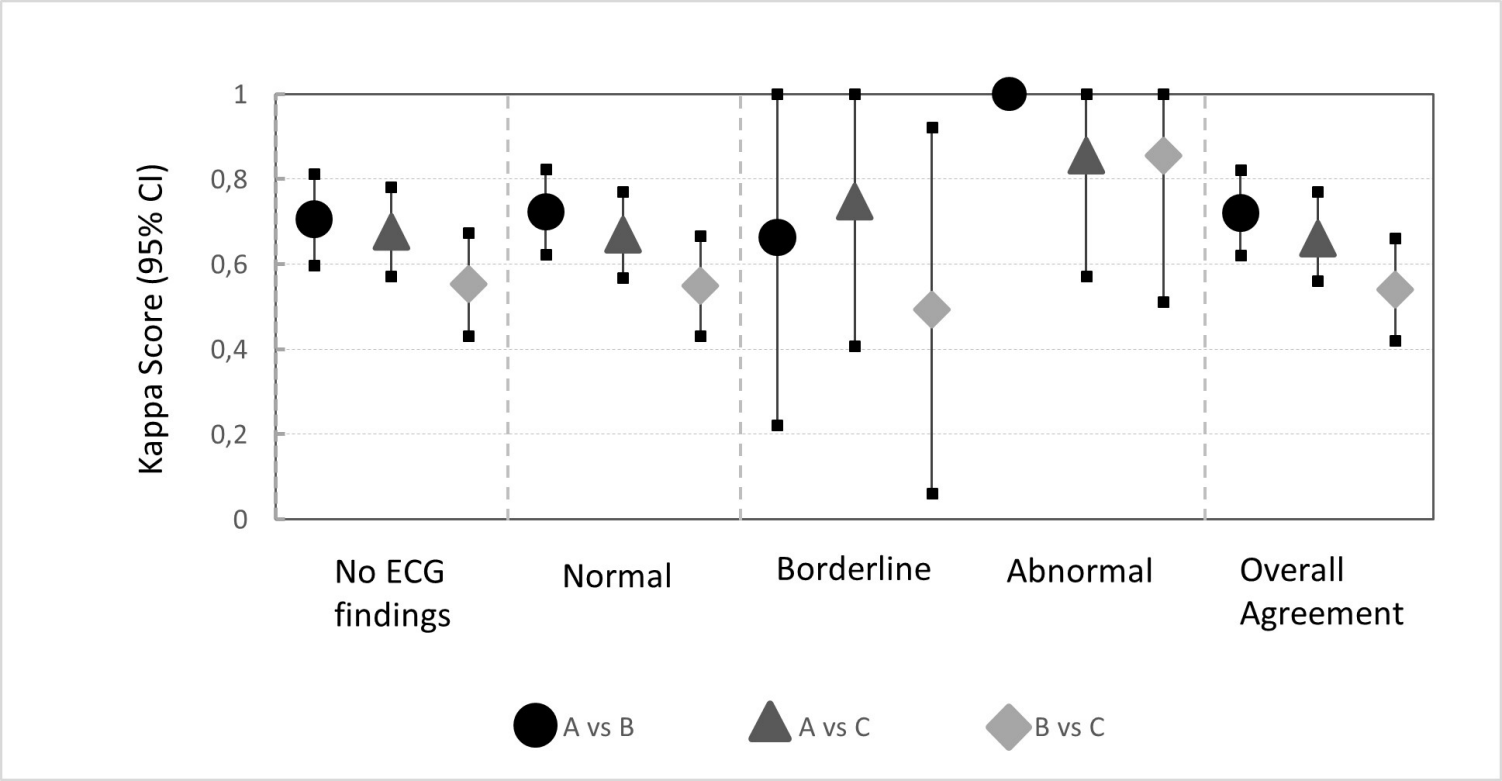
κ agreement for no, normal in athletes, borderline and abnormal ECG findings according to the recent International criteria. CI, confidence interval.

κ agreement for no, normal in athletes, borderline and abnormal ECG findings and agreement overall according to the recent International recommendations is shown in Fig 2.

## Discussion

The present pilot investigation is the first to assess the inter- observer agreement in athlete ECG interpretation of consecutive Swiss elite athletes according to the recently published International recommendations. [9–11] We demonstrated an excellent inter-observer agreement in the detection of abnormal ECG findings among observers with different levels of expertise after a specific training in athlete ECG interpretation. ECG interpretation resulted in a moderate to good overall agreement.

### The importance of using standardized criteria and the level of experience in ECG interpretation

**Fig 3:**
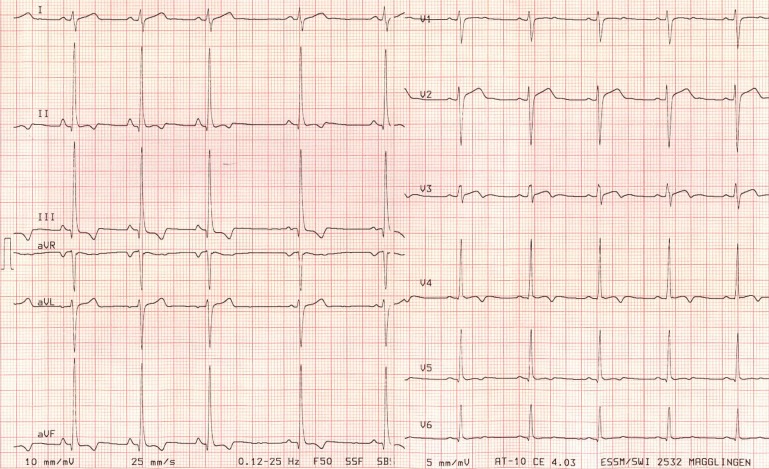
Resting ECG of a 17-year old triathlete with infero-lateral TWI and findings suggestive of a former myocarditis during cardiologic work-up.

It has been consistently shown that using standardized criteria improves the accuracy of the ECG interpretation among competitive athletes. Drezner et al. found an improved sensitivity and specificity after using a standardised interpretation tool based on the 2010 original ESC criteria in a small sample of pathology-enriched ECGs (12 out of 40 with cardiac pathology) and a broad variety of expertise among 60 different interpreters (primary care residents, attending physicians, sports medicine physicians, cardiologists).[13] Thereafter, Exeter et al. showed that the use of standardised criteria improved the accuracy of ECG findings even in less experienced physicians.[12] In fact, they presented a total of 40 ECGs including 10 ECGs that were pathology-enriched athlete ECGs to 31 physicians familiar with the standardized interpretation tool (intervention group) and to 31 physicians not familiar with the tool (control group). Using the online standardised criteria tool lead to a reduction in the false-positive ratings. Hill et al. showed in a small sample of pathology-enriched ECGs (8 out of 18 with cardiac pathology) of a pediatric athletic population a high inconsistency in the accurate diagnosis among 53 pediatric cardiologists, which resulted in more additional testing and higher rates of inappropriate sports guidance compared to experts (2 electrophysiologists who had 100% concordance for all diagnoses). [21] Interestingly, there was no significant association between the correct ECG interpretation and the degree of experience in their study. The first prospective study addressing this issue in a real-world setting (i.e. non-enriched with known pathological ECGs) consisted of 440 consecutive PPS ECGs of asymptomatic elite athletes. With regard to the presence of pathological findings, Brosnan et al. found only fair to moderate agreement between three different but experienced interpreters (a sports cardiologist, a sports medicine physician and an electrophysiologist) using the 2010^th^ ESC criteria. [4] These results were partly confirmed by Berte and co-workers in another prospective cohort of young Belgian soccer players. [5] This was notably the first study not only to show a decrease in the prevalence of abnormal ECGs but also a higher overall agreement using the more recent original Seattle criteria compared to the 2010^th^ ESC criteria, particularly among cardiologists. Using the refined Seattle criteria, Sheikh et al. showed in a retrospective sub-analysis of their seminal paper on 1000 randomly selected athlete ECGs an excellent inter-observer agreement between the first and senior authors, both with a high level of expertise in athlete ECG interpretation (κ score of 0.97).[22] The same study group recently compared the 2010^th^ ESC criteria, the original and refined Seattle criteria among eight cardiologists (4 inexperienced and 4 experienced in athletes screening, both groups consisting of 3 general cardiologists and one electrophysiologist). κ agreement for abnormal findings was moderate for the refined criteria among both groups (κ scores of 0.41 and 0.43 for inexperienced and experienced cardiologists, respectively) with an improvement among inexperienced cardiologists compared to the older screening criteria. [17] Comparing our results between the most recent International recommendations with the older standardized screening criteria (i.e. 2010 original ESC criteria, original Seattle criteria), all TWI would have been detected as pathological finding with all the different screening criteria. Only the athlete with a borderline Q wave duration would have been potentially missed with the 2010 ESC criteria. Remarkably, two observers in our pilot investigation had little or no expertise in the athlete ECG interpretation, but were specifically trained according to the most recent International recommendations.[9–11] This approach resulted in an excellent inter-observer agreement in the detection of abnormal ECG findings which require further cardiologic work-up. No ECG abnormality was missed by neither of the two observers with less expertise. All three observers identified the 3 identical male athletes with TWI. Only one of the 3 athletes with TWI as an abnormal ECG finding revealed significant structural heart disease during further cardiologic work-up. The 17-year old triathlete with infero-lateral TWI showed a small mid-wall scar in the inferior-apical segment in the cardiac MRI, suggestive of a former myocarditis (Fig 3). Transthoracic echocardiography performed before had revealed a segmental reduction in left ventricular longitudinal strain. Neither of the two remaining athletes with isolated TWI (1 male swimmer, 1 male triathlete) presented echocardiographic findings associated with a clear underlying cardiomyopathy.

### The impact of inter-observer agreement on downstream cardiologic work-up

**Fig 4:**
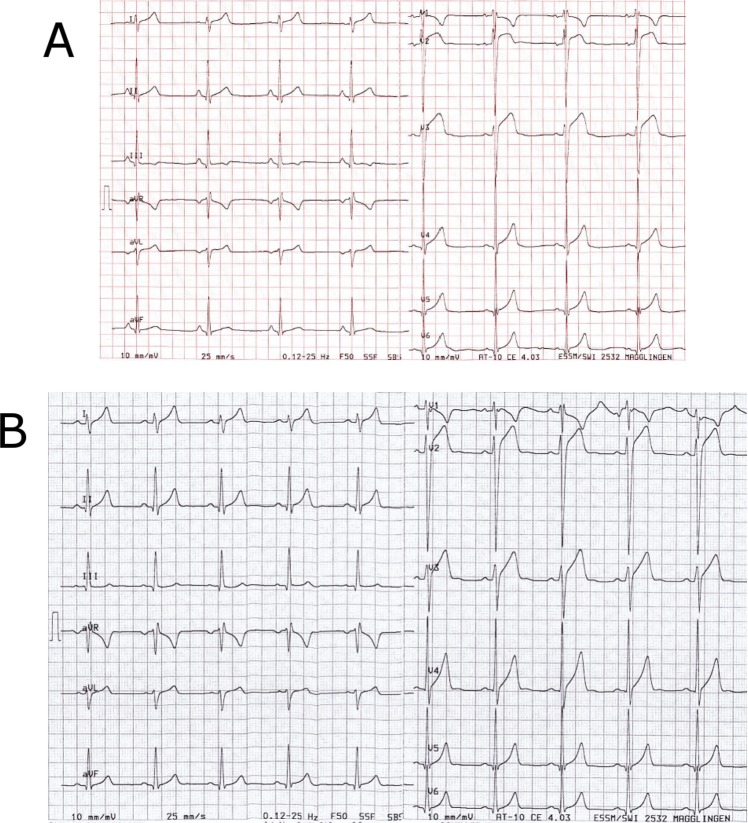
Resting ECG of 24-old cyclist with a pathological Q wave duration in lead V4-V6 at baseline (a) and a normal Q wave duration 4 months later (b).

In their study with 400 consecutive athlete ECGs, Dhutia et al. recently found that inexperienced cardiologists were 5 times more likely to refer an athlete for further cardiologic work-up based on ECG findings.[3] Moreover, they calculated a 2-fold increase in cost for ECG-based screening by inexperienced cardiologists compared to their experienced counterparts. However, in our study both observers with less expertise classified only one additional ECG as abnormal with regard to the recent International recommendations compared to our reference (observer C). Namely, observer C interpreted one ECG with a borderline Q-wave duration of 46–50 ms in V4-V6 visually as normal while observer A and B measured with manual calipers (Fig 4A). The 24-year old cyclist declined a further work-up. The follow-up ECG 4 months later showed no abnormal ECG findings indicating the initial ECG was a false-positive finding (Fig 4B). This case may also support the necessity of follow-up ECGs in case of abnormal ECG findings.

In summary, our pilot investigation emphasizes that a specific training in athlete ECG interpretation based on the recent standardized diagnostic criteria (i.e. International recommendations for ECG interpretation in athletes) can improve inter-observer agreement, even in observers with little or no expertise in the athlete ECG interpretation. Moreover, since ECG-based cardiovascular pre-participation screening of athletes is endorsed by the European Society of Cardiology, this study supports the need for providing a specific training in ECG interpretation for physicians involved in pre-participation screening. [1, 2] This may help to improve the inter-observer agreement in athlete ECG interpretation and therefore reduce the rate of downstream cardiologic work-up.

### Limitation

The results of our study have to be interpreted in the light of several limitations. The number of abnormal ECG findings in our cohort of consecutive Caucasian athletes was very low, but comparable to a cohort of U.S. college athletes. [23–25] Remarkably, more than one third of athletes in our cohort were female, which might have significantly affected our rate of ECG pathology. The higher number of abnormal ECGs found in other studies may be explained by their design, using pathology-enriched ECGs, [12, 13, 21] the older 2010^th^ ECG interpretation algorithm with known higher prevalence of abnormal findings, [4, 5, 17, 22] a random sample with young male soccer players with different races, [5] predominantly male athletes participating in high dynamic sports disciplines [4, 23], and finally, a possible selection bias of athletes evaluated at international expert centers. [17, 22] Inter-observer agreement may be lower in cohorts with mixed ethnicities and a larger variety of abnormal ECG patterns, requiring higher expertise of ECG interpretation. Therefore, our findings cannot be extended to the ECG interpretation of athletes of black and/or Arab race. Finally, the results provided in our study cannot be generalized, particularly not to observers which are not specifically trained in athlete ECG interpretation.

## Conclusions

Our cohort of elite athletes had a low prevalence of abnormal ECGs. Agreement in abnormal ECG findings was excellent in unexperienced but trained observers. ECG interpretation with the recent International recommendations for ECG interpretation in athletes resulted in an acceptable overall agreement among observers with different levels of expertise.

## Supporting information

S1 File Raw data of all analysed data. (SAV)
